# Measuring expansion from macro‐ to nanoscale using NPC as intrinsic reporter

**DOI:** 10.1002/jbio.201900018

**Published:** 2019-05-28

**Authors:** Luca Pesce, Marco Cozzolino, Luca Lanzanò, Alberto Diaspro, Paolo Bianchini

**Affiliations:** ^1^ Nanoscopy and NIC Department, Istituto Italiano di Tecnologia Genoa Italy; ^2^ Department of Physics University of Genoa Genoa Italy

**Keywords:** expansion microscopy, nanoscopy, nucleoporin, stimulated emission depletion microscopy, super‐resolution

## Abstract

Expansion microscopy is a super‐resolution method that allows expanding uniformly biological samples, by increasing the relative distances among fluorescent molecules labeling specific components. One of the main concerns in this approach regards the isotropic behavior at the nanoscale. The present study aims to determine the robustness of such a technique, quantifying the expansion parameters i.e. scale factor, isotropy, uniformity. Our focus is on the nuclear pore complex (NPC), as well‐known nanoscale component endowed of a preserved and symmetrical structure localized on the nuclear envelope. Here, we show that Nup153 is a good reporter to quantitatively address the isotropy of the expansion process. The quantitative analysis carried out on NPCs, at different spatial scales, allows concluding that expansion microscopy can be used at the nanoscale to measure subcellular features with an accuracy from 10 to 5 nm. Therefore, it is an excellent method for structural studies of macromolecular complexes.
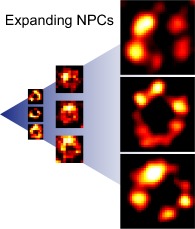

## Graphical abstract

Imaging NUPs at different expansion factors, the images are acquired using STED nanoscopy.

## 1 INTRODUCTION

Expansion microscopy (ExM) is a comparatively new approach to super‐resolution imaging with conventional fluorescence microscopy 1, 2, 3, 4. It is based on the physical enlargement of a sample to increase distances between spatially unresolved objects. The Abbe law states that two objects can be resolved if they are at a distance larger than the diffraction limit. Instead of optically circumventing such a limit, Boyden and co‐workers developed a method to artificially increase the relative distance among objects, so that they become resolvable. The availability of hydrogels, with the property of absorbing large amount of water by swelling and increasing the occupied volume by approximately 90‐fold 1, makes the expansion of a fixed biological specimen feasible. When the expansion is uniform in the three dimensions and the fluorescence is preserved, the final imaging resolution is improved by the same factor. A 4‐fold expansion means a spatial resolution of about 40 to 50 nm in a standard confocal laser scanning microscope operating with a high, >1, numerical aperture (NA) objective. Such a resolution is typical for super‐resolution techniques like stimulated emission depletion (STED) 5, 6, 7, saturated structured illumination (SSIM) 8, and photoactivated localization microscopy (PALM) 9. Since ExM generally preserves the fluorescence, we can apply a super‐resolved method like STED to the expanded sample achieving a further notable increase in spatial resolution 10, 11. The immediate result using STED is that an additional resolution gain of 2‐ to 3‐fold can be reached at low depletion power, also suggesting the parallelization of the process can be achieved towards high throughput imaging 12.

It is self‐evident that a key challenge in ExM is to verify the isotropy of the expansion process and to quantify the expansion of biological structures, correlated to the gel size. In general, the evaluation of the expansion factor (EF) is a delicate step of the method. It is required for each expanded sample and the option of measuring the gel size, before and after the expansion, at a macroscale, may be not accurate enough to quantify expansion at all scales. Indeed, the precise evaluation of the EF is not only the difficulty by itself but appears to be hampered by possible distortions and heterogeneities. Processes like gelation and digestion can alter the positions of the cross‐linked fluorescent labels and introduce artifacts in the distribution and organization of proteins in the cells. For these reasons, it is extremely important to investigate the native conformation of macromolecular complexes before and after expansion.

Up to now, the most sensitive method to compare and quantify the expansion factor requires imaging the labeled sample before and after expansion. In particular, it needs to identify the same cells and the same focal plane before and after the process. Thus, it requires the acquisition of three‐dimensional (3D) large field of view at the highest resolution possible, typically implemented by stitching (3D) stacks acquired on a confocal laser scanning microscope. Such a method is time consuming and could increase the photo‐bleaching, limiting the photons budget after expansion. A good compromise between speed and resolution is offered by the spinning disk confocal microscope, which has been used for the imaging of microtubules in the pre‐ and post‐expanded samples 1. Unfortunately, the sensitivity of these methods is limited to the scale of few hundreds of nanometers and they cannot be used to quantify expansion properties below this spatial scale.

To overcome such a limitation, ExM has been combined with other super‐resolution techniques. Indeed, the combination of ExM with other super‐resolution methods, for example, ExM‐STED (ExSTED) 11, UltraExM STED 14, and SIM‐ExM 13, has been recently used to show the isotropic expansion for cytoplasmic structures, such as the cytoskeleton, and symmetric macromolecular complexes like the centrosomes 11, and centrioles 14. Such solutions enabled a resolution of about 10 nm 10, 11, suggesting its relevance to quantify the isotropic expansion at the nanoscale level. However, the overall fluorescence photon budget can be reduced by recurring and high illumination imposing a new strategy. Nevertheless, quantification of 2d expansion process has also been carried out using fluorescent DNA nanorulers by Scheible and Tinnefeld 15.

Our strategy is to use a highly preserved and well‐characterized macromolecular structure as intrinsic reporter to evaluate and measure the expansion process. Such molecular assembly could facilitate the calculation of the EF and its distortion, avoiding any mapping of the biological specimen. The nuclear pore complex (NPC) is the target and the key actor of the method. Thanks to electron and super‐resolution optical microscopy, a precise description of the NPC structure is available 16, 17. It is characterized by an outer diameter of approximately 100 nm, a central transport channel of 40 nm and can be precisely localized in the nuclear envelope. Its structure is composed by 8‐fold rotational symmetry consisting of a cytoplasmic and nuclear ring connected by scaffold proteins built around a central channel. Each side of this channel is associated with eight filament subunits, where they form a highly ordered structure called “nuclear basket” inside the nucleus 18.

**Figure 1:**
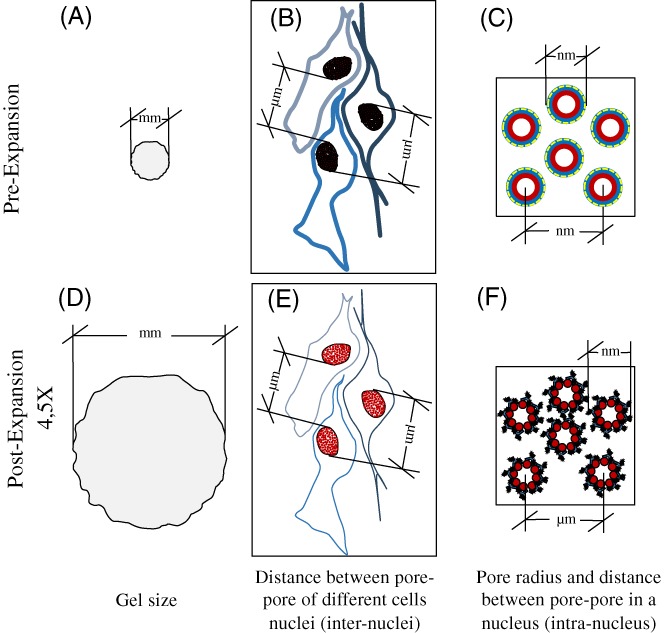
Schematic representation of the correlation between macro‐, micro‐ and nano‐scale expansion of the sample labeled for nuclear pore complex (NPCs). The top and the bottom row show a scheme of pre‐expansion (Pre)‐ and post‐expansion (Post‐Ex) samples and the strategy to confirm the isotropy and the expansion factor (EF) at different magnitude. At the macroscale level, measuring the gel size Pre‐ (A), post‐digestion (Post‐Dig) (not showed) and Post‐Ex (D). At the microscale level, quantifying the distance between pores in a same nucleus (intra‐nucleus) and/or different nuclei (inter‐nuclei) in Pre‐ (B, C) and Post‐Ex (E, F); and, finally, at the nanoscale level, applying a Pre‐ (C), Post‐Dig (not showed) and Post‐Ex (F) quantitative radius analysis on NPC. The microscale analysis requires the same cells labeled for NPCs before and after expansion, while at the nanoscale is not necessary

Here, we show that a combination of ExM and STED nanoscopy on the NPC can be used to verify the isotropy of the expansion at the nanoscale. Thanks to the capability of ExSTED to resolve fine details of biomolecular assemblies, particle averaging 16, 19 is performed to confirm an 8‐fold symmetrical arrangement of the Nup153 subunit in the pore after expansion. This strategy allows to apply a quantitative pre‐expansion (Pre‐Ex), post‐digestion (Post‐Dig) and post‐expansion (Post‐Ex) analysis, defining the nanoscale expansion and the distortion for biological structures. At the same time, the hydrogel properties can also be evaluated at the microscale and macroscale level, by measuring the pore‐to‐pore distances and the gel size, respectively (Figure 1).

## 2 MATERIALS AND METHODS

### 2.1 Cell culture and immunostaining

Chozinski et al proposed a variant of the originals ExM in which the samples are labeled with conventional antibodies 3. We adapt our STED labeling protocol with the ExM proposed by Chozinski. In general, for super‐resolution techniques and in particular for ExM, a labeling density using antibodies is critical for the final resolution and artifact‐free images 23.

Hos and Hek cells are grown in Dulbecco's Modified Eagle Medium (DMEM) supplemented with 10% fetal bovine serum and 1% pen/strep and glutamine. For nuclear pore labeling, immunofluorescence is carried out using an adopted protocol from Szymborska et al 16. Hos cells are plated at 70% confluency on 18 mm coverglass and grown overnight. The cells are pre‐extracted with 2.4% PFA and 0.3% Triton‐X100 in PBS for 3 min. After fixation with 2.4% PFA for 30 min, the cells are blocked for 1 hour with 5% BSA. Then, the cells are incubated overnight at 4°C with primary antibody (Nup153, ab84872 from AbCam) in BSA 5%. After washing several times in PBS, the cells are incubated with the secondary antibody at room temperature for 1 hour. Figure S1 shows two different antibodies used to confirm the ring‐like structure of Nup153 subunit.

For α‐Tubulin (T5168 from Sigma), Hek cells are pre‐extracted with 0.25% glutaraldehyde and 0.3% Triton‐X100 for 3 minutes and successively fix with 3.2% PFA 0.25% glutaraldehyde in PBS for 10 minutes. After rinsing with PBS and blocking with 5% BSA and 0.3% Triton‐X100 for 1 hour, the sample is incubated overnight at 4°C with primary antibody. After washing several times in PBS, the cells are incubated with the secondary antibody at room temperature for 1 hour. To link antibodies and endogenous proteins to the gel, the sample, labeled for NPC and α‐Tubulin, is rinsed in 25 mM Methacrylic acid *N*‐hydroxy succinimidyl ester (MA‐NHS) for 60 minutes at room temperature.

### 2.2 Polymerization, digestion and expansion

The samples are soaked in a gelation solution of 2 M NaCl, 2.5% (Wt/Wt) acrylamide, 0.15% (Wt/Wt) *N*,*N*′‐methylenebisacrylamide, 8.625% (Wt/Wt) sodium acrylate, 0.2% (Wt/Wt) tetramethylene diamine (TEMED) and 0.2% (Wt/Wt) ammonium persulfate (APS) in DI water, with the APS added last. The salinity concentration and the ratio between acrylamide and *N*,*N*′‐methylenebisacrylamide determine a mesh pore size of 1 to 2 nm 1. The gelation solution (~50 μL) is placed on the hydrophobic surface, and the coverglass is placed on top of the solution with cells face down. Gelation is allowed to proceed at 37°C for 20 minutes. The coverglass and gel are removed with tweezers and placed in digestion buffer (1× TAE buffer, 0.5% Triton X‐100 and 0.8 M guanidine HCl) containing 8 units mL^−1^ proteinase K added freshly. Gels are digested overnight. To finalize the sample preparation and analyze it, the gel is removed from digestion buffer and placed on a cover glass and imaged at the confocal and STED microscope. After imaging, the hydrogel is moved into 60 mm petri dish and soaked in ~50 mL DI water to expand it. Water is exchanged every 30 minutes and 4 times until expansion is complete. At the final expansion, small gel stripes are cut and observed by confocal and STED microscopy.

### 2.3 Fluorescence microscopy

Both pre‐, post‐Dig and post‐Ex NPC imaging are obtained using a commercial Leica TCS SP5 gated STED‐CW microscope (Leica Microsystems, Mannheim, Germany) with a 100×, 1.4 NA oil immersion objective. The excitation and depletion wavelengths are 488 and 592 nm respectively, with a collection spectral window of 510–560 nm. Spinning disk confocal microscope (Nikon Eclipse T*i* coupled with Andor Revolution XD) is used to map a small portion of the gel before expansion using large image modality, with a 20×, NA 0.75 dry objective (Figure S2, A). To visualize nuclei and α‐tubulin labeled with Hoechst 33342 and Atto647N, the excitation wavelengths used are 405 and 640 nm, respectively. To see the position and borders of the hydrogel, we acquired the transmission image too. After mapping different areas, specific cells in different region are acquired using confocal Leica TCS SP5 microscope, visualizing Nup153 and α‐tubulin labeled with Alexa Fluor 488 and Atto647N, with a 100×, 1.4 NA oil immersion objective (Figure S2, B and C). To reduce hydrogel drift during microscope acquisition, Poli‐L‐lysine (Sigma) is applied on 24 mm round #1.5 coverglass for 30 min at 37 °C. After, the expanded gel is soaked in a solution of 2% agarose to immobilize it. The same cells acquired in the pre‐expansion modality are imaged after expansion using Nikon A1 Confocal Microscope with a 60× Obj, NA 1.4 oil immersion objective. Due to the 3D expansion it is challenging to find the same focus. Thus, different stacks are acquired for the basal portion of the nuclei and successively aligned and summed by Fiji 24. After cropping an area of interest, the images are normalized using Fiji.

### 2.4 Imaging processing and data analysis

For the radius analysis, single pores are manually cropped with a diameter of 0.2, 0.4 and 0.9 μm using Fiji. The radius of each pore is calculated using a custom Matlab script. This algorithm calculates multiple radial intensity profiles along different orientations. For each orientation, the radial distance between the maximum value of the profile and the center is calculated. Finally, the radius of the pore is calculated as the average of these radial distances. In order to obtain an average value and SD for each sample, we fit the histogram obtained from all the analyzed images with a Gaussian distribution. In the case of the ExSTED sample, for each pore, we also generate an angular intensity profile, by plotting the maximum value of the radial intensity as a function of the orientation. In order to obtain the angular position of the subunits, we fit the angular intensity profile with a multi‐Gaussian peaks function. Peaks with a width larger than a given threshold are discarded to reject unresolved subunits (Figure S6). Peaks with an amplitude lower than a given threshold are discarded to reject unspecific labeling. For each pore, the angular position of the first detected peak is subtracted from all the detected peak angle values. In other words, the first detected subunit is used as a reference (angle = 0°). The cumulative histogram relative to the angle values retrieved from the analysis more than 100 pores is fitted with a 7 peaks‐Gaussian fit. Finally, for the microscale analysis, the Pre‐ and Post‐ex image registrations are carried out using the affine image registration in the TurboReg plugin 25 (Figure S3, C), which cuts, translates and rotates to overlap the images. The Fast 2D Peak Finder script (Version 1.12.0.0, Natan, 11 Oct 2013) is used to calculate the distortion error (Figure S3).

## 3 RESULTS

### 3.1 Visualization of NPCs labeled for Nup153 at different expansion factors and optical resolutions

Our goal is to investigate and compare the extent of expansion at different spatial scales. Figure 1 shows a scheme of what we can measure before and after expansion. At the millimeter scale, we can measure the gel size (Figure 1 A, D), at micrometer scale, we can measure the distances between nuclei (Figure 1B,E) and NPCs (Figure 1C, F) and finally, at the nanoscale, we can measure size and shape descriptors of the NPCs (Figure 1C, F). The well‐preserved structure of the NPC makes the calculation of the expansion factor and the distortion at the nanoscale easier, because does not require to map the same NPC before and after expansion. In particular, our attention is focused on a specific subunit called Nup153, localized on the nucleoplasmic side. Indeed, Walther et al reported a precise localization of Nup153 at the base of the nuclear basket by immunogold labeling, with a distance from the centre of the channel of 47.0 ± 8.8 nm 20.

In this work, we monitor three check points in the expansion process: after gelation, after digestion, and after dialysis in MilliQ water. Although expansion starts already after digestion, the expansion process reaches its maximum—about 5‐fold—only at the last step. An advantage of using a STED microscope is that we can easily tune the optical resolution, for instance by changing the temporal gating and/or the depletion power 21. Therefore, we can keep a constant overall resolution at different expansion levels. It immediately results in a reduction of photo‐bleaching: more expansion, less STED power, less photo‐bleaching, better signal‐to‐noise ratio. However, it is worth noting that, in both ExM and STED, the number of fluorescent molecules is reduced by a factor that depends on the reduction of the effective observation volume.

**Figure 2:**
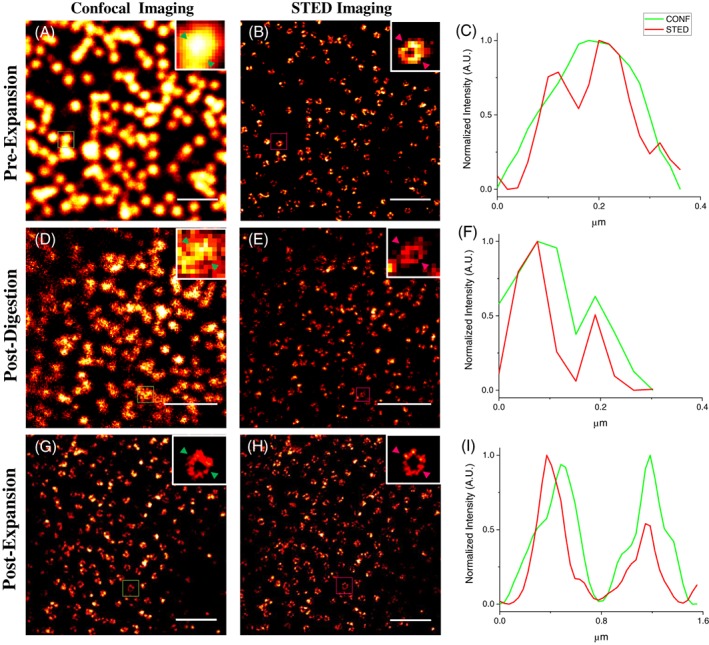
Nuclear pore complex (NPCs) visualized at various expansion factors and optical resolutions. Different conditions that can tune the final resolution in an expansion microscopy (ExM)/stimulated emission depletion (STED) experiment, including the expansion factor, the diameter of the pinhole, gating and power of the STED beam are considered. (A) Pre‐expansion (Pre‐Ex) confocal imaging, excitation (Exc) at the sample 13.3 μW; (B) Pre‐Ex STED Imaging, STED power at the sample 72.2 mW, Gating 2 to 9.5 ns, Pinhole 0.6 AU. Scale bar Pre‐Ex: 1 um; (D) Post‐digestion (Post‐Dig) confocal imaging, Exc at sample 13.3 μW; (E). Post‐Dig STED Imaging, STED power at the sample 41 mW, Gating 1.5 to 9.5 ns, Pinhole 0.6 AU. EF ~2x, scale bar; (G) Post‐Ex confocal imaging, Exc at the sample 13.3 μW; (H) Post‐Ex STED imaging, STED power at the sample 27 mW, Gating 1 to 9.5 ns, Pinhole 1 AU. EF ~4×, scale bar: 1 μm; (C, F, I) Line profiles of selected NPCs

In particular, in Figure 2 we show an example of NPC imaging at the three check points. We compare confocal and STED imaging. While confocal imaging on pre‐Ex sample shows a spotty image where the empty basket is not resolved and many NPCs appear to form large aggregates (Figure 2A), STED imaging before expansion (Figure 2B) and confocal imaging after expansion (Figure 2, G) clearly show the ring shape of the NPCs at comparable resolution. The Post‐Dig sample shows intermediate characteristics in terms of signal and resolution (Figure 2A‐D) respect to the pre‐ and post‐ex specimens. However, we noticed a worse imaging quality than the other check points, maybe caused by the presence of the digestion buffer in the gel that could quench the fluorescence. At the maximum expansion, confocal microscopy allows observing the characteristic ring‐like structure (Figure 2G). From a qualitative analysis, we can gather that expansion is isotropic. It confirms that ExM is an excellent tool to investigate molecular structures in any area of the cell. However, to see the single subunits, we need to use STED nanoscopy (Figure 2H).

It is worth noting that, in this case, where the sample is expanded, the power of the STED laser is much reduced respect to the Pre‐Ex case (Figure 2B). It means that photo‐bleaching is reduced and signal‐to‐noise ratio is improved. Therefore, different EF and the possibility to tune the optical resolution play an important role in the final resolution achievable.

### 3.2 The expanded Nup153 has octagonal symmetry

As we qualitatively shown in Figure 2, the ring‐like structure of the NPC becomes clearly visible at different expansion folds and confocal resolution (Figure 2). However, only using ExSTED it is possible to resolve finer details and distinguish single Nup153 subunits (Figure 2H). We now aim to demonstrate the 8‐fold symmetry of Nup153 in the Post‐Ex samples, by means of a quantitative analysis on ExSTED images of the NPCs.

**Figure 3:**
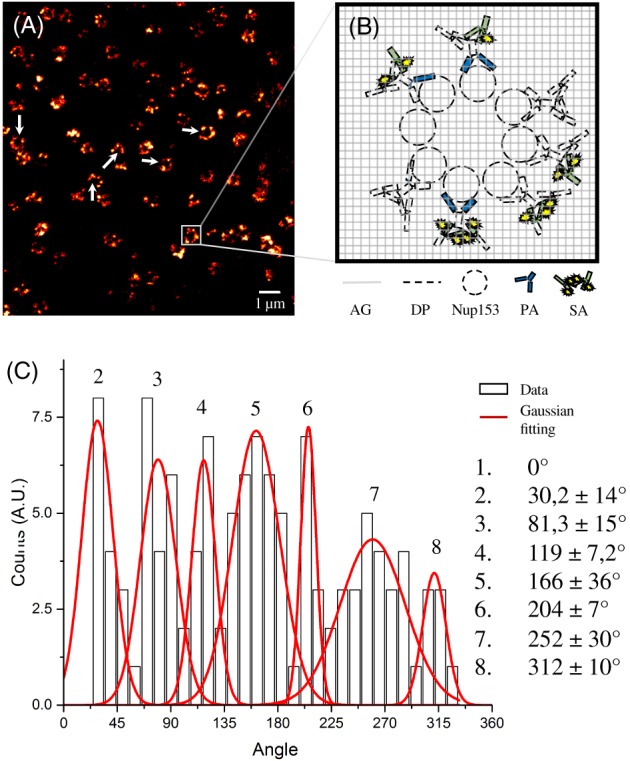
After expansion, Nup153 preserves its octagonal symmetry. As a consequence of the antibodies used, the polymerization and the digestion process, we have a loss of fluorescent signal from the Nup153 subunits. It is evident in (A) (white arrows), in which the sample is characterized by a wide heterogeneity. (B) Shows a schematic illustration what happens in a single nuclear pore complex labeled for Nup153 soaked in the gel after expansion. A way to overcome these limitations and clarify the octagonal structure of Nup153 after expansion is particle averaging. The histogram obtained (C) is characterized by seven peaks, corresponding to the angular difference between the reference subunit (imposed to 0^°^) and the other subunits. As shown, the angular difference among each subunits is a multiple of about 45^°^ , demonstrating the pore octagonal symmetry of Nup153 subunit after expansion. GA, gel network; DG, digested proteins; PA, primary antibodies; SA, secondary antibodies

Collecting a large quantity of nuclear pore images, we notice a wide sample heterogeneity (Figure 3A). The defective labeling of some structures, visible as open rings, could be due to the digestion process or the staining heterogeneity. In fact, although we follow a labeling protocol for super‐resolution imaging, gel polymerization and the digestion process may induce a final fragmented labeling for several Nup153 subunits and a loss of fluorescence signal (Figure 3B). To overcome these limitations, we use a statistical approach. From the ExSTED images acquired at the highest resolution, we isolate more than a hundred of single NPCs. For each NPC, we localize the Nup153 subunits and calculate their angular distance with respect to a subunit taken as reference (see Material and Methods). A multi‐peaks fit of the cumulative histogram shows an angular difference among the subunits—7 peaks respect to the reference subunit (imposed to 0°)—that is a multiple of 45° (Figure 3C). This analysis thus confirms the eightfold symmetry and disposition of the subunits (Figure 3C). This result shows that STED enables a quantitative analysis at the nanoscale in expanded samples, allowing us to define single Nup153 subunits and confirm an eightfold symmetrical arrangement of the ring.

### 3.3 Nanoscale and macroscale quantification of the expansion process

**Figure 4:**
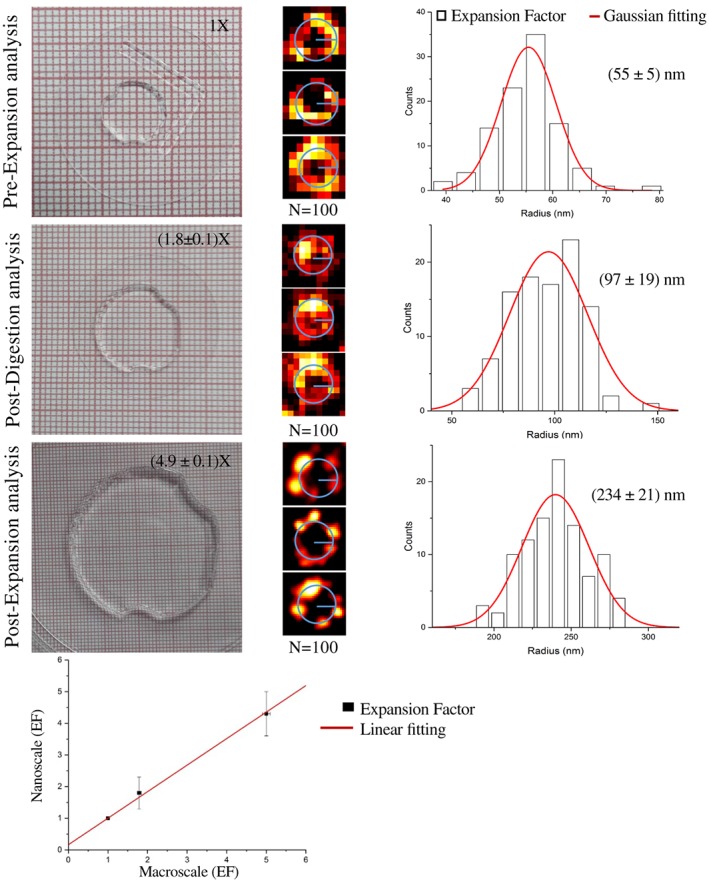
Quantitative pre‐expansion (Pre‐Ex), post‐digestion (Post‐Dig) and post‐expansion (Post‐Ex)/stimulated emission depletion (STED) analysis. Macroscopic diameter of the Pre‐, Post‐Dig (EF = 1.79 ± 0.05) and Post‐Ex (EF = 5 ± 0.1) hydrogel. (B, E, H) Examples of nuclear pore complexes (NPCs) which are selected at different expansion time and imaged by STED nanoscopy. (C, F, I) The histograms show the quantitative Pre‐, Post‐Dig and Post‐Ex analysis of n = 100 NPCs and the medium radius obtained. The EF of the pore radius obtained is 1.8 ± 0.5 after digestion (EF_Post‐Dig_) and 4.3 ± 0.7 after expansion (EF_Post‐Ex_). (L) shows the good correlation between the nanoscale and the macroscale EF

We quantify the EF at the nanoscale by measuring the radius of the pores. We image Nup153 by means of STED nanoscopy (Figure 2B) at the three check points, that is, Pre‐Ex, Post‐Dig and Post‐Ex. As in the previous analysis, we manually select more than hundred pores (Figure 4B, E and H) to quantify their radius and shape. In keeping with Walther et al 20, the measured radius of the ring labeled for Nup153 in the Pre‐Ex sample is (55 ± 5) nm. Since the ratio of the size of the gel before and after digestion is 1.79 ± 0.05 (Figure 4D, D), we consider the Post‐Dig sample as intermediate expansion. The measured pore radius in this sample is (96 ± 19) nm that corresponds to an EF_Post‐Dig_ of 1.8 ± 0.5 (Figure 4F).

In the last step of expansion method, consisting in MilliQ water dialysis, the obtained macroscopic expansion of the gel is 5 ± 0.1 (Figure 4G). The pore radius measures (234 ± 21) nm, which correspond to an EF_Post‐Ex_ of 4.3 ± 0.7 (Figure 4I). Figure 4L, shows that the measures of expansion factors obtained on the gel, at the macroscale and on the pores, at the nanoscale, are in good correlation.

Knowing the precise expansion factor at the nanoscale clarifies the final achievable resolution for the three different check points. The temporal gating, the depletion power and the pinhole size are modulated as a function of the expansion degree (Figure 2) and permit us to tune the spatial resolution. The improvement of resolution provided by STED at any given experimental condition is calculated using the method reported in Reference 21. In the Pre‐Ex sample, the optical conditions allow us to obtain a resolution of about 54 nm (Figure 2, Figure S5). Obviously, an increase of the gel size corresponds to the reduction of the STED intensity, gating time and pinhole opening. In the Post‐Dig sample, the estimated optical resolution of the STED microscope is 74 nm (Figure S5), which combined with an expansion factor EF_Post‐Dig_ = 1.8 yields a final effective resolution of 74 nm/EFPost‐Dig = 41 nm. In the Post‐Ex sample, the resolution obtained is 100 nm, which combined with an expansion factor EF_Post‐Ex_ = 4.3 yields a final effective resolution of 100 nm/ EF_Post‐Ex_ = 23 nm.

### 3.4 Microscale quantification and distortion of the expansion process

Finally, we measure the distance between pores localized on the same nucleus and on nuclei of different cells. Since it requires imaging the same cells before and after the expansion, this procedure is remarkably demanding in terms of time and light exposure. For this reason, it is not possible to acquire the specimens with STED nanoscopy and we skip the Post‐Dig samples in this analysis. The Pre‐ and Post‐Ex stitched confocal images are rescaled by affine registration (Figure S2, D; Figure S3). These measurements are performed mapping eight different cells labeled for NPC and tubulin (Figure S2). In particular, tubulin labeling helps in the identification of cells before and after expansion but is not used in the registration process. Using the pixel size ratio between Post‐ and Pre‐Ex imaging, resulting from this image analysis, we measure an EF of 3.8 ± 0.1 from the intra‐nuclei pore‐to‐pore distances and 4.3 ± 0.4 from the inter‐nuclei pore‐to‐pore distances (Table 1). We note that, in this last group, the measured EF seems dependent on the cell localization. In these samples, Hek cells are arranged in clusters divided by empty region of hydrogel. Such a heterogeneous distribution of cells is reflected in the measured expansion factors at different scales, that is, the inter‐nuclei expansion factor measured within a cell cluster is lower than the same factor measured from cells not belonging to the same cell cluster. Although these differences in EF on average are small locally, they can be relevant as shown in Figure S4. It demonstrates that the expansion process can be heterogeneous at the microscale depending on the local properties of the specimen.

**Table 1:** Correlation between nano‐, micro‐ and macroscale expansion

Expansion Factor (X)				
		Microscale		
	Nanoscale	Intra‐nucleus	Inter‐nuclei	Macroscale
Post‐dlgestion	(1.8 ± 0.5)X	‐	‐	(1.79 ± 0.05)X
Post‐expansion	(4.31 ± 0.7)X	(3.8 ± 0.1)X	(4.3 ± 0.4)X	(5.0 ± 0.1)X

The table synthetizes the expansion values obtained using the quantitative analysis at different EF and scale. For the microscale validation, we apply an affine registration on single cells nuclei and cellular clustered nuclei to determine the distance between the pores (intra‐ and inter‐nuclei analysis). The data obtained correlate with the other expansion factors.

## 4 DISCUSSION AND CONCLUSION

In this work, we explore the effects induced in a conserved nuclear structure, that is, NPC, by soaking in a swellable polymer network. We used such a symmetric structure as a reporter to verify the isotropy and precisely quantify the expansion of biological structures. The risk of distortions that the expansion process could produce on complex molecular assemblies, is a potential problem of this technique and it must be deeply investigated. Therefore, we implemented a quantitative approach, that taking advantage of STED imaging, reaches the resolution able to verify the arrangement of molecules embedded in the expanded hydrogel. As described, we refer to the published electron microscopy studies 20, when we measure the localization and the radius of Nup153 in the NPC. We found only a few studies about this specific subunit, which are in agreement with our results.

Regarding the achievable signal, the indirect immunofluorescence is one of the best methods to label NPCs. Although there are several other labeling approaches, for example, Nanobody antibodies 22, they often suffer for degradation during the digestion and/or crosslink failure to the hydrogel 11, limiting their use for ExM experiments.

After demonstrating the 8‐fold symmetry of Nup153 after expansion, we apply a quantitative analysis to measure the pore radius, verifying the distortion due to the gelation and expansion at the nanoscale level. This analysis confirms that the relative error does not change in the Pre‐ and Post‐Ex samples (9%). The pore size variability is due to the heterogeneity of the labeling for NPCs, but not to the distortion induced by gelation and expansion. In comparison, the Post‐Dig sample is characterized by a high error value (20%), probably due to incomplete and not isotropic expansion.

These data are confirmed by the pre‐ and post‐Ex pixel size ratio between pores belong to the same nucleus or to adjacent nuclei (microscale analysis, Table 1). Finally, we distinguish two different expansion factor for neighboring cells at microscale level (Figure S4). Our hypothesis to explain it is that the hydrogel without biological sample is characterized by higher EF values (space between not connected cells, expansion factor of 4.6 ± 0.1), while the incomplete digestion in some cell clusters can cause resistance to the expansion (3.9 ± 0.2; Figure S4).

In summary, we find that NPCs expand uniformly and ExM technique results suitable for nuclear protein structures. To demonstrate it, we introduce different approaches, which are ExSTED, particle averaging and registration in expanded samples. Obviously, this does not exclude that, for other cellular elements, the native structures can be distorted or generate spotted artifact at molecular level. For this reason, it is necessary to examine in depth the behavior of other molecular complexes, proteins and, as well, chromatin‐DNA in expanded samples. However, we do not find any evidence for sudden alterations in individual super‐resolved molecular structures, demonstrating the possibility to use NPC as universal tool to reveal uniformity and the real expansion value for biological samples. ExSTED is a promising tool for the study of molecular structure at the nanoscale.

## Supporting information


**FIGURE S1.** Hos cells labeled for Nup153 with Alexa 488. Labeling Hos cells with two different antibodies for Nup153 (Figure A Sigma (HPA027897), Figure B AbCAM (ab84872)), we obtained the same ring‐like structure using STED nanoscopy.
**FIGURE S2.** Mapping method of the soaked sample in the hydrogel for microscale analysis. The expansion factor at the microscale level and the distortion are measured by overlapping and rescaling the confocal images of the same cell before and after expansion. In this way, we analyze the right distribution of NPCs at the microscale level. The expansion factor is calculated by the ratio of the Post‐Ex and Pre‐ pixel size in confocal images; these measurements are performed several times on each sample (8 times). The triangular shape of the hydrogel, α‐tubulin and DNA labeling allows mapping the same cell before and after expansion. **(**A) Shows a mosaic image using Spinning Disk. The image is an overlay of the transmission channel (shown in gray look‐up table, LUT), and two fluorescent channels, DNA labeled with Hoechst 33342 (shown in blue LUT) and tubulin labeled with Atto647N channel (shown in red LUT). After this mapping, a specific area is selected and imaged using confocal microscopy (tubulin only in red LUT) (B). A cell is selected and imaged at Nyquist sampling as shown in (C). With the aim of comparing pre‐ and post‐expansion images only tubulin (red LUT) and Nup153 (green LUT) channels are acquired and overlaid, (C) and (D). After expansion, the same cells of (C) are imaged using confocal microscopy (D).
**FIGURE S3.** Calculating of the expansion and distortion factor by affine registration. (A) and (B) show a confocal Pre‐ and Post‐Ex imaging of the same nucleolus labeled for NPC with Nup153. (C) Shows the affine registration between the two images using TurboReg (Fiji). This strategy allows us to obtain the expansion factor by pixel size ratio and the distortion error, calculating the distance pore to pore (red circle: Nup pre‐expansion; green cross: Nup post‐expansion). After registration, we use the Fast 2D Peak Finder script (Version 1.12.0.0, Natan, 11 Oct 2013), which allows the maximum relative values to be found, corresponding to the NPCs of the Pre‐ and Post‐Ex. The distance between pores and an estimation of the percentage error are calculated (D).
**FIGURE S4.** The microscale analysis demonstrates an heterogenic expansion factor. The affine registration between Pre‐ (red) and Post‐Ex (green) images shows a heterogenic expansion. Indeed, the inter‐nuclei EF measured from cells belonging to the same cluster is 3.9 ± 0.2, while the inter‐nuclei EF calculated from cells not belonging to the same cell cluster is (4.6 ± 0.1). We cannot record the third cells (on the top right), because the EF is higher respect to the clustered cells (measure performed with four different cells).
**FIGURE S5.** Estimation of the resolution of the Pre‐, Post‐Dig and Post‐Ex samples combined to STED. The depletion curves are obtained by acquiring linearly five frames by increasing the STED power, from the confocal (I_1_) to the STED maximum (I_5_). The conditions used are reported in the main text. The frames shown in Figure A, B and C are acquired on a Pre‐Ex sample, corresponding to a maximum of the STED power of 27 mW, 41 mW and 72.2 mW, respectively. From the average depletion curves we extracted the k parameter necessary to calculate the improvement of the resolution of the different check points. Approximating the confocal resolution (R_conf_) of about 250 nm and dividing for the nanoscale expansion factor, we obtain a final resolution (R_f_) of 23 nm, 41 nm and 54 nm in the Post‐ex, Post‐Dig and Pre‐Ex, respectively. Scale bar 1 μm.
**FIGURE S6.** Confirming octagonal symmetry of Nup153. Example of analysis performed in a single pore. Using the algorithm described in Materials and Methods, we fit the angular profile using a multi‐Gaussian peaks function in order to obtain the angular position of the subunits for each peaks. Moreover, we discard the peaks with wide angular values (e.g., subunit 2, red peak) and low amplitude, to reject unresolved subunits (eg, subunit 2) or aspecific labeling. We repeated this analysis for each pore we selected (N ˃ 100).
