# Supplementary material for: Measuring expansion from macro‐ to nanoscale using NPC as intrinsic reporter
Source: J Biophotonics. 2019 May 28;12(8):e201900018. doi: 10.1002/jbio.201900018 (PMC7065622; doi:10.1002/jbio.201900018)
Supplement: Supplementary file 1 — FIGURE S1. Hos cells labeled for Nup153 with Alexa 488. Labeling Hos cells with two different antibodies for Nup153 (Figure A Sigma (HPA027897), Figure B AbCAM (ab84872)), we obtained the same ring‐like structure using STED nanoscopy. FIGURE S2. Mapping method of the soaked sample in the hydrogel for microscale analysis. The expansion factor at the microscale level and the distortion are measured by overlapping and rescaling the confocal images of the same cell before and after expansion. In this way, we analyze the right distribution of NPCs at the microscale level. The expansion factor is calculated by the ratio of the Post‐Ex and Pre‐ pixel size in confocal images; these measurements are performed several times on each sample (8 times). The triangular shape of the hydrogel, α‐tubulin and DNA labeling allows mapping the same cell before and after expansion. (A) Shows a mosaic image using Spinning Disk. The image is an overlay of the transmission channel (shown in gray look‐up table, LUT), and two fluorescent channels, DNA labeled with Hoechst 33342 (shown in blue LUT) and tubulin labeled with Atto647N channel (shown in red LUT). After this mapping, a specific area is selected and imaged using confocal microscopy (tubulin only in red LUT) (B). A cell is selected and imaged at Nyquist sampling as shown in (C). With the aim of comparing pre‐ and post‐expansion images only tubulin (red LUT) and Nup153 (green LUT) channels are acquired and overlaid, (C) and (D). After expansion, the same cells of (C) are imaged using confocal microscopy (D). FIGURE S3. Calculating of the expansion and distortion factor by affine registration. (A) and (B) show a confocal Pre‐ and Post‐Ex imaging of the same nucleolus labeled for NPC with Nup153. (C) Shows the affine registration between the two images using TurboReg (Fiji). This strategy allows us to obtain the expansion factor by pixel size ratio and the distortion error, calculating the distance pore to pore (red circle: Nup pre‐expan [file JBIO-12-e201900018-s001.docx]

((Supporting Information should be submitted in a separate file))

Supporting Information

Measuring expansion from macro- to nanoscale using NPC as intrinsic reporter

Luca Pesce, Marco Cozzolino, Luca Lanzanò, Alberto Diaspro and Paolo Bianchini^1*^

Supplementary Figures


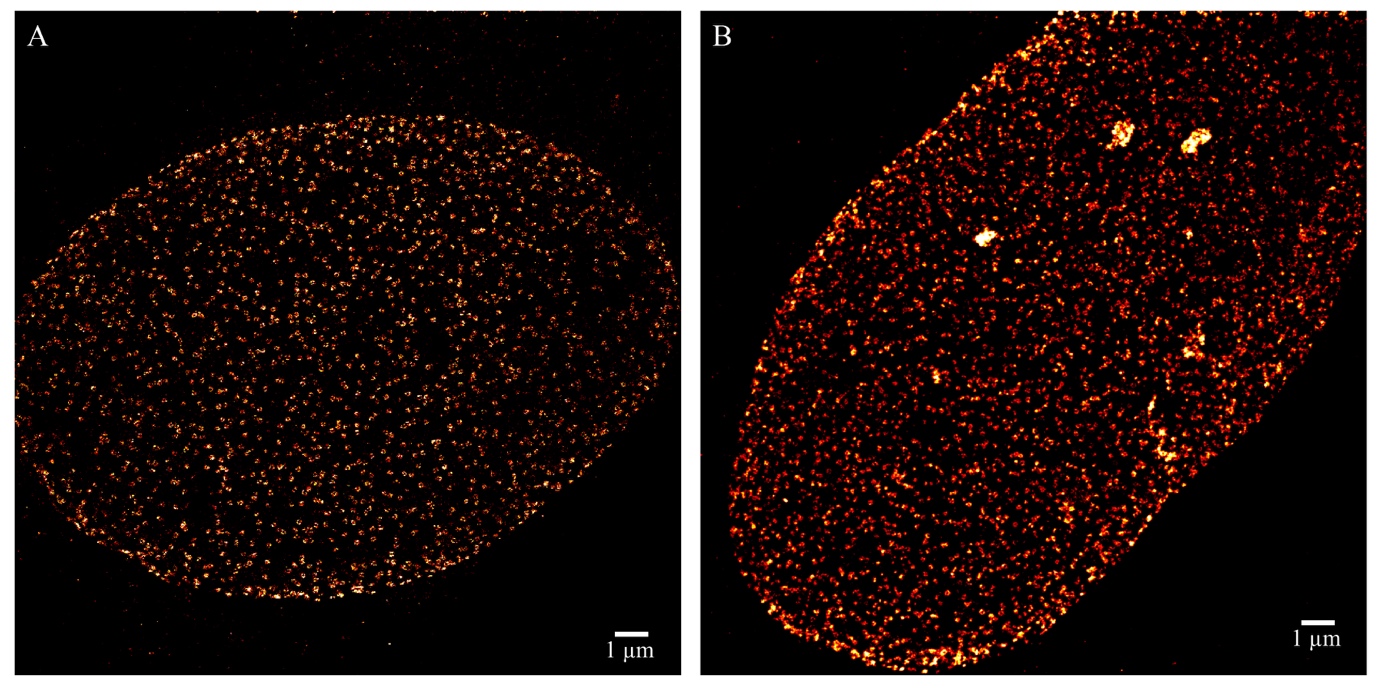


**Figure S1: Hos cells labelled for Nup153 with Alexa 48**8. Labelling Hos cells with two different antibodies for Nup153 (Fig. A Sigma (HPA027897), Fig. B AbCAM (ab84872)), we obtained the same ring-like structure using STED nanoscopy.


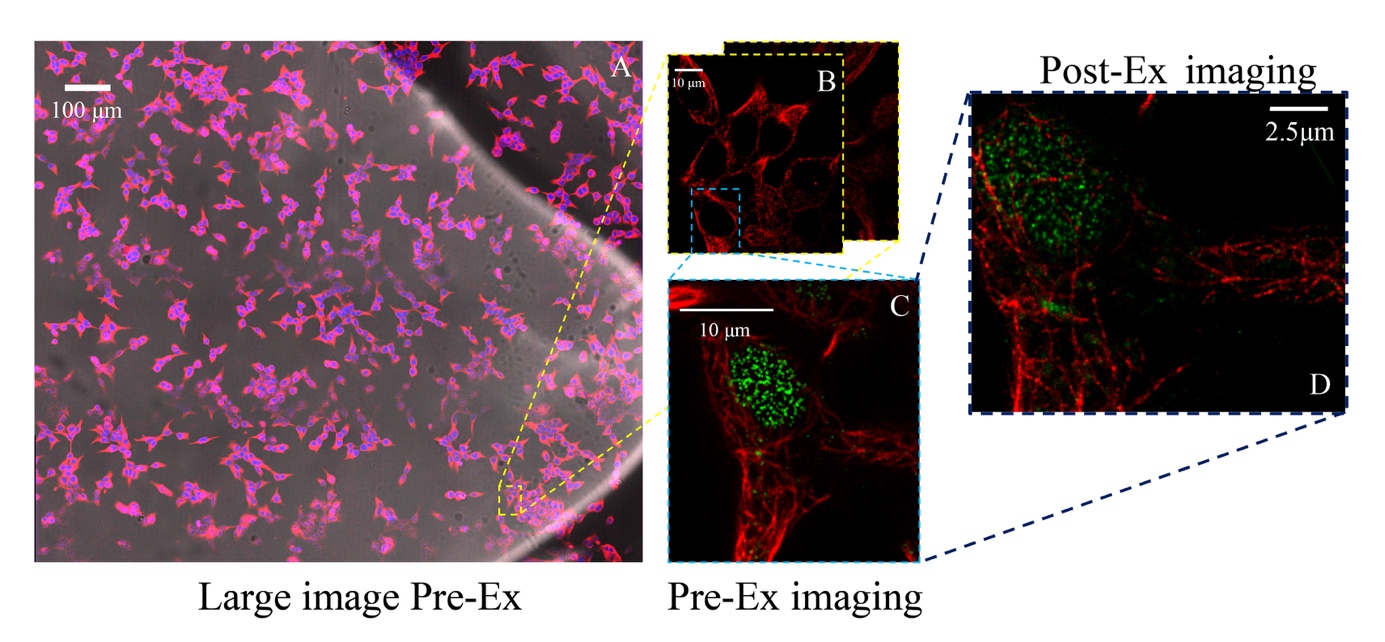


**Figure S2. Mapping method of the soaked sample in the hydrogel for microscale analysis.** The expansion factor at the microscale level and the distortion are measured by overlapping and rescaling the confocal images of the same cell before and after expansion. In this way, we analyze the right distribution of NPCs at the microscale level. The expansion factor is calculated by the ratio of the Post-Ex and Pre- pixel size in confocal images; these measurements are performed several times on each sample (8 times). The triangular shape of the hydrogel , α-tubulin and DNA labelling allows mapping the same cell before and after expansion. **(A)** Shows a mosaic image using Spinning Disk. The image is an overlay of the transmission channel (shown in grey look-up table, LUT), and two fluorescent channels, DNA labeled with Hoechst 33342 (shown in blue LUT) and tubulin labeled with Atto647N channel (shown in red LUT). After this mapping, a specific area is selected and imaged using confocal microscopy (tubulin only in red LUT) (**B**). A cell is selected and imaged at Nyquist sampling as shown in **(C).** With the aim of comparing pre- and post-expansion images only tubulin (red LUT) and Nup153 (green LUT) channels are acquired and overlaid, **(C)** and **(D)**. After expansion, the same cells of **(C)** are imaged using confocal microscopy (**D**).


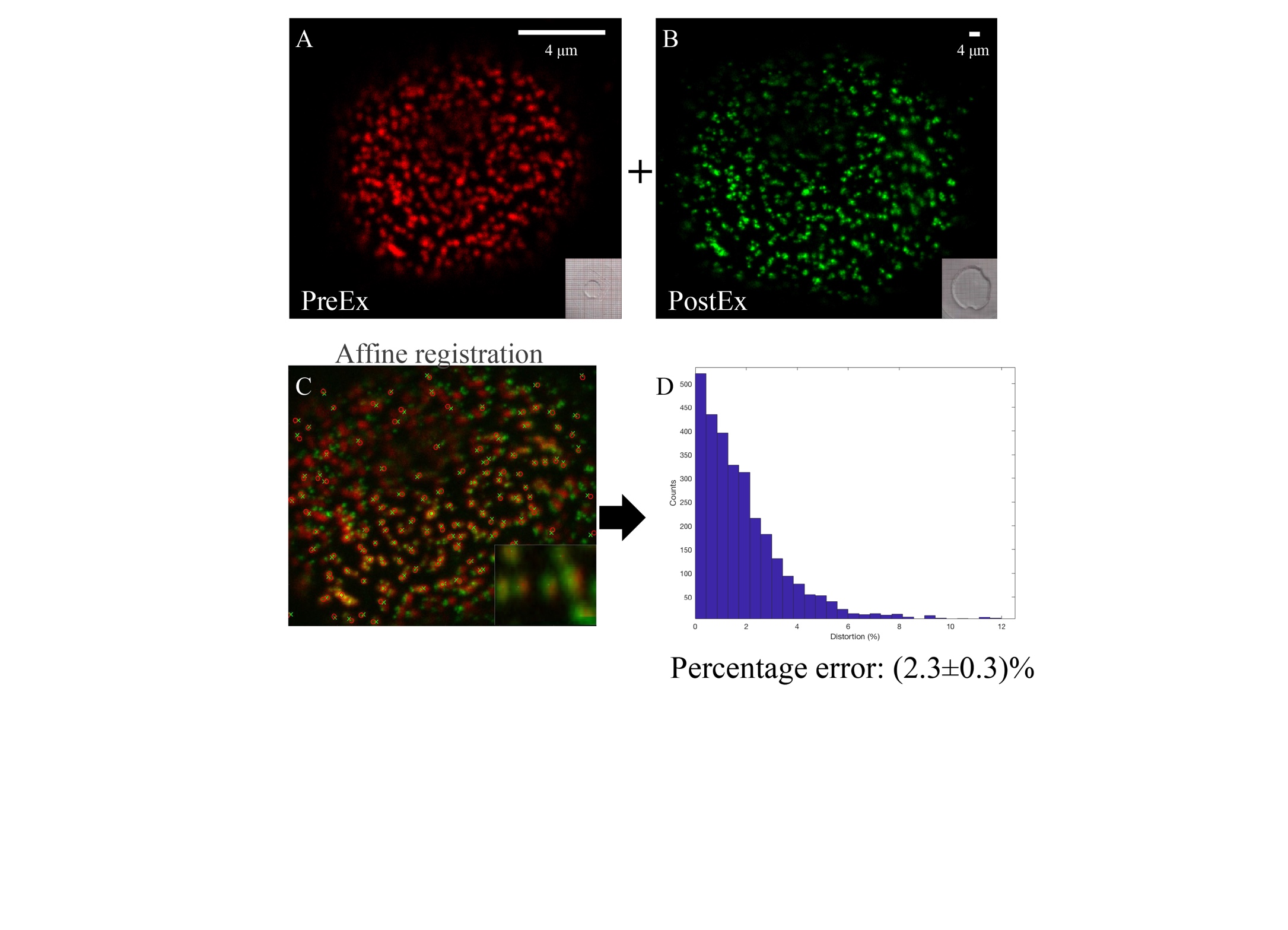


**Figure S3. Calculating of the expansion and distortion factor by affine registration. (A)** and (**B)** show a confocal Pre- and Post-Ex imaging of the same nucleolus labelled for NPC with Nup153. (**C)** shows the affine registration between the two images using TurboReg (Fiji). This strategy allows us to obtain the expansion factor by pixel size ratio and the distortion error, calculating the distance pore to pore (red circle: Nup pre-expansion; green cross: Nup post-expansion). After registration, we use the Fast 2D Peak Finder script (Version 1.12.0.0, Natan, 11 Oct 2013), which allows the maximum relative values to be found, corresponding to the NPCs of the Pre- and Post-Ex. The distance between pores and an estimation of the percentage error are calculated (**D**).


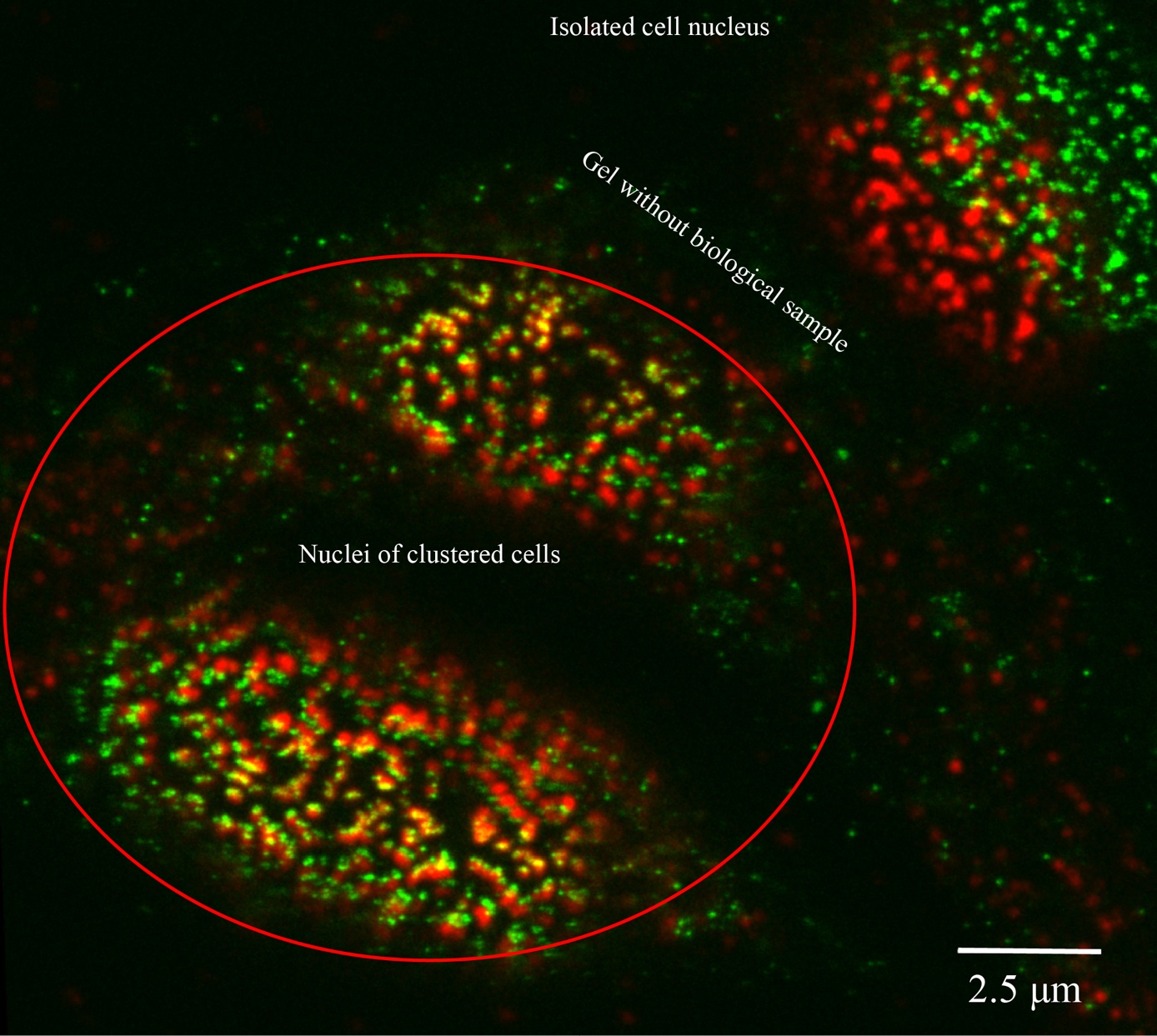


**Figure S4. The microscale analysis demonstrates an heterogenic expansion factor.** The affine registration between Pre- (red) and Post-Ex (green) images shows a heterogenic expansion. Indeed, the inter-nuclei EF measured from cells belonging to the same cluster is 3.9 ± 0.2, while the inter-nuclei EF calculated from cells not belonging to the same cell cluster is (4.6 ± 0.1). We cannot record the third cells (on the top right), because the EF is higher respect to the clustered cells (measure performed with four different cells).


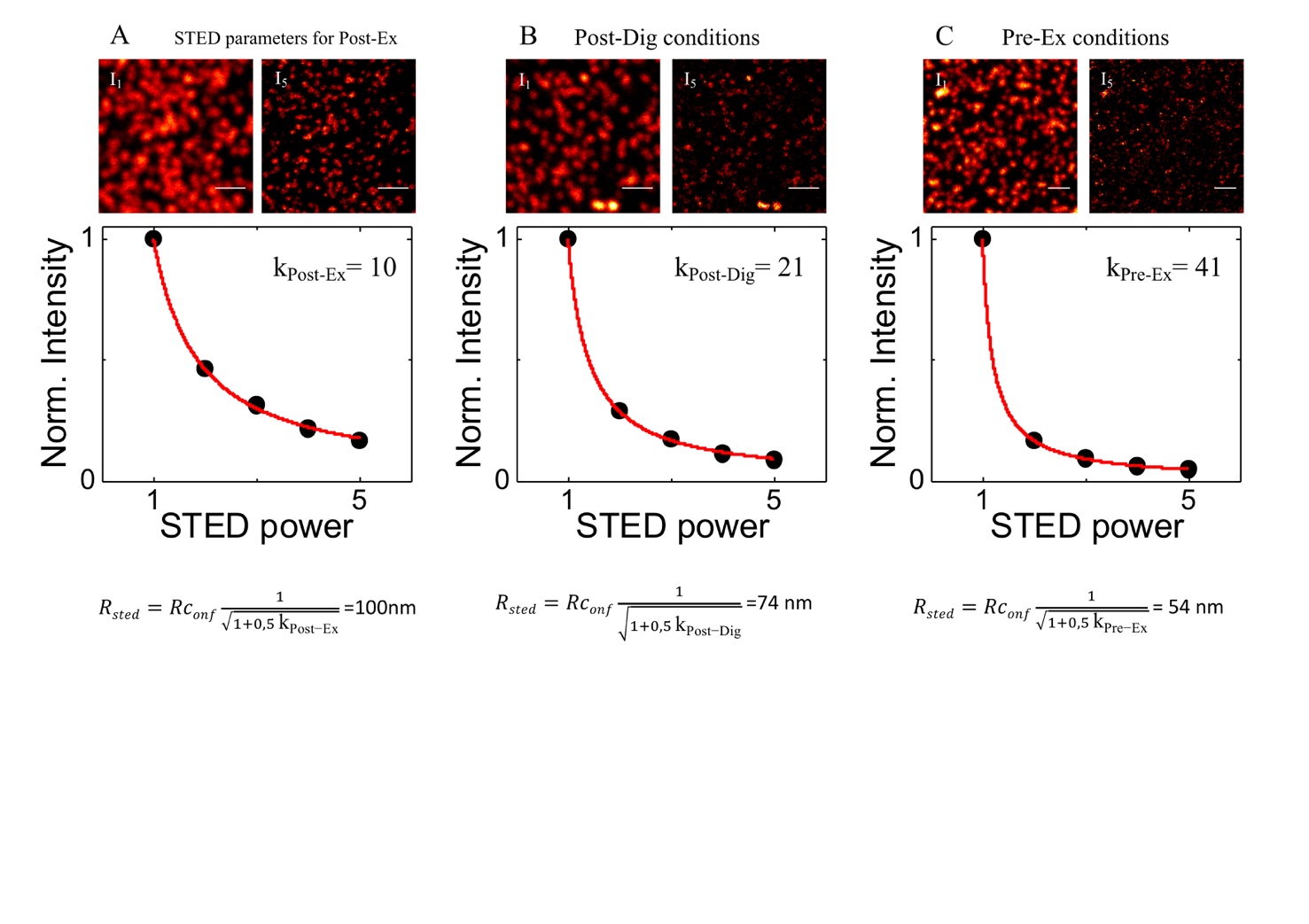


**Figure S5. Estimation of the resolution of the Pre-, Post-Dig and Post-Ex samples combined to STED.** The depletion curves are obtained by acquiring linearly five frames by increasing the STED power, from the confocal (I_1_) to the STED maximum (I_5_). The conditions used are reported in the main text. The frames shown in Fig. A, B and C are acquired on a Pre-Ex sample, corresponding to a maximum of the STED power of 27 mW, 41 mW and 72.2 mW, respectively. From the average depletion curves we extracted the k parameter necessary to calculate the improvement of the resolution of the different check points. Approximating the confocal resolution (R_conf_) of about 250 nm and dividing for the nanoscale expansion factor, we obtain a final resolution (R_f_) of 23 nm, 41 nm and 54 nm in the Post-ex, Post-Dig and Pre-Ex, respectively. Scale bar 1 μm.


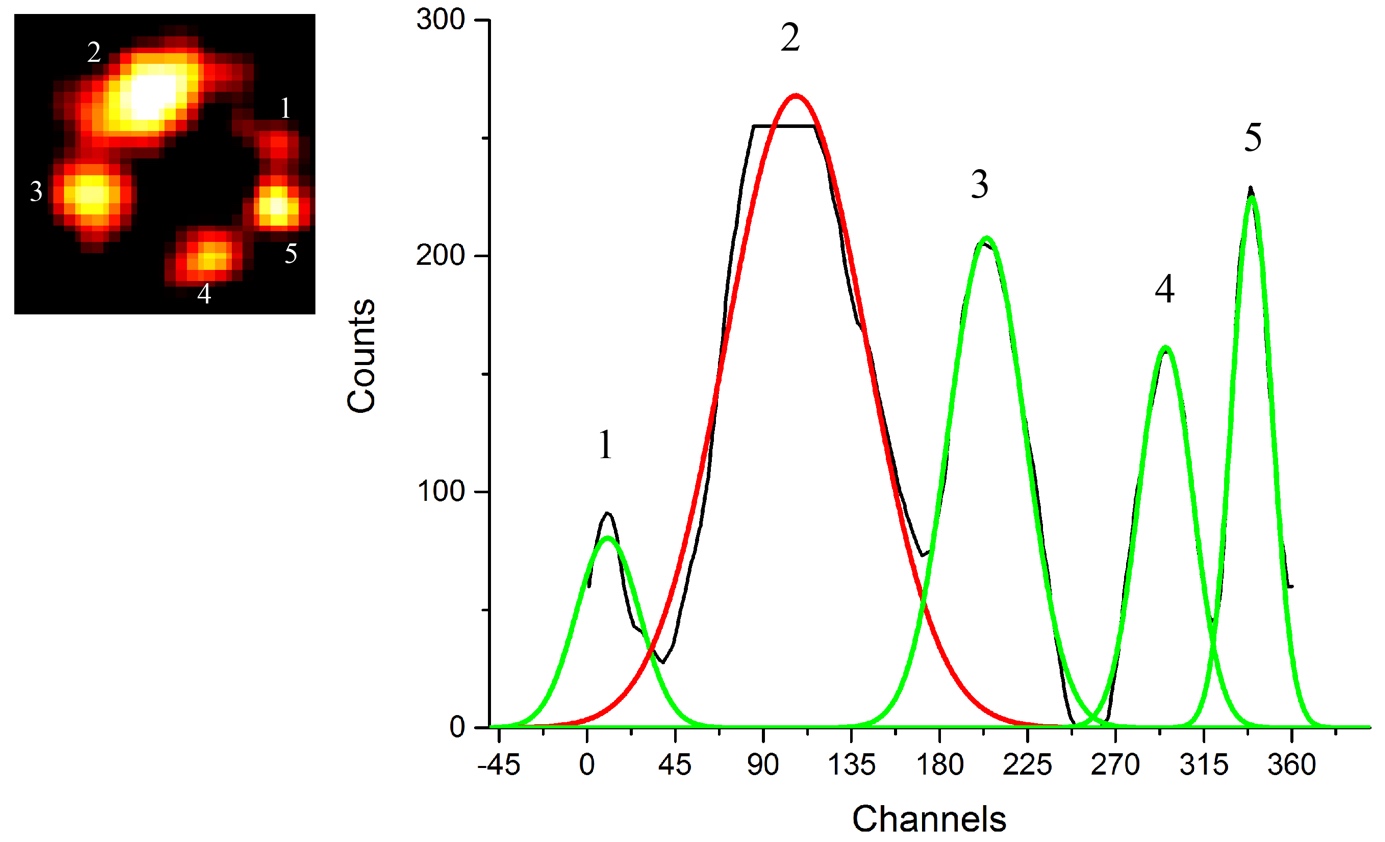


**S6. Confirming octagonal symmetry of Nup153.** Example of analysis performed in a single pore. Using the algorithm described in Materials and Methods, we fit the angular profile using a multi-Gaussian peaks function in order to obtain the angular position of the subunits for each peaks. Moreover, we discard the peaks with wide angular values (e.g., subunit 2, red peak) and low amplitude, to reject unresolved subunits (e.g., subunit 2) or aspecific labeling. We repeated this analysis for each pore we selected (N˃100).
